# Preliminary feasibility assessment of CDM-based active surveillance using current status of medical device data in medical records and OMOP-CDM

**DOI:** 10.1038/s41598-021-03332-6

**Published:** 2021-12-15

**Authors:** Sooin Choi, Soo Jeong Choi, Jin Kuk Kim, Ki Chang Nam, Suehyun Lee, Ju Han Kim, You Kyoung Lee

**Affiliations:** 1 Department of Laboratory Medicine and Genetics, Center for Medical Device Safety Monitoring, Soonchunhyang University College of Medicine, Soonchunhyang University Bucheon Hospital, 170 Jomaru-ro, Bucheon, 14584 Republic of Korea; 2 Department of Internal Medicine, Center for Medical Device Safety Monitoring, Soonchunhyang University College of Medicine, Soonchunhyang University Bucheon Hospital, 170 Jomaru-ro, Bucheon, 14584 Republic of Korea; 3 Department of Medical Engineering, Dongguk University College of Medicine, Gyeongju, 38066, Republic of Korea; 4 Department of Biomedical Informatics, College of Medicine, Konyang University, Daejeon, 35365 Republic of Korea; 5 Division of Biomedical Informatics, Seoul National University Biomedical Informatics (SNUBI), Seoul National University College of Medicine, Seoul, 08826 Republic of Korea

**Keywords:** Health care, Medical research, Computational biology and bioinformatics

## Abstract

In recent years, there has been an emerging interest in the use of claims and electronic health record (EHR) data for evaluation of medical device safety and effectiveness. In Korea, national insurance electronic data interchange (EDI) code has been used as a medical device data source for common data model (CDM). This study performed a preliminary feasibility assessment of CDM-based vigilance. A cross-sectional study of target medical device data in EHR and CDM was conducted. A total of 155 medical devices were finally enrolled, with 58.7% of them having EDI codes. Femoral head prosthesis was selected as a focus group. It was registered in our institute with 11 EDI codes. However, only three EDI codes were converted to systematized nomenclature of medicine clinical terms concept. EDI code was matched in one-to-many (up to 104) with unique device identifier (UDI), including devices classified as different global medical device nomenclature. The use of UDI rather than EDI code as a medical device data source is recommended. We hope that this study will share the current state of medical device data recorded in the EHR and contribute to the introduction of CDM-based medical device vigilance by selecting appropriate medical device data sources.

## Introduction

Medical devices play important roles in disease diagnosis, prevention, and treatment. However, they also carry potential risks of serious injuries and even fatality^1,2^. Therefore, postmarket medical device vigilance (MDV) is crucial for public health protection, and medical device adverse event (MDAE) information should be collected, evaluated, analyzed, and disseminated through a timely and reliable method^3^. Current MDV methods rely on passive reporting, which is a combination of mandatory and voluntary adverse event reporting systems used by patients, physicians, manufacturers, and healthcare organizations. For a long time, these reporting systems have been useful for identifying unexpected and unique adverse events^4^. However, passive surveillance is limited by the voluntary nature of reporting, the strong inherent bias associated with spontaneous reports, underreporting, and the lack of denominator data on comprehensive exposure^4–7^. To overcome these limitations, an active surveillance system via common data model (CDM) could be helpful. CDM is a logical and semantic data model that can be used to standardize multiple data sources into a common format. It has been effectively implemented with pharmaceutical products (including vaccines)^8^. In addition, applying passive and active surveillance simultaneously using electronic health records (EHR) could augment sample size, increase population heterogeneity, and cross-validate results^9^.

In recent years, there has been an emerging interest in the use of claims and EHR data for evaluating medical device safety and effectiveness^10^. Attempts have been made to implement MDV active surveillance through a web-based platform for sharing the experiences on medical device incidents^11^ and through a multicenter, prospective, observational research study^12^. However, both studies used a centralized method with patient data entered into the database as a common language agreed between network organizations. Studies of CDM-based MDV have not been reported yet. There is a difference between pharmacovigilance and medical device. Pharmacovigilance uses CDM to analyze adverse events associated with the use of a drug while medical device vigilance analyzes adverse events associated with the use of a medical device. This study was conducted to identify the current situation and derive improvements, assuming that medical device information deficiencies in EHR or CDM could become a hurdle for CDM-based MDVs.

## Results

**Figure 1:**
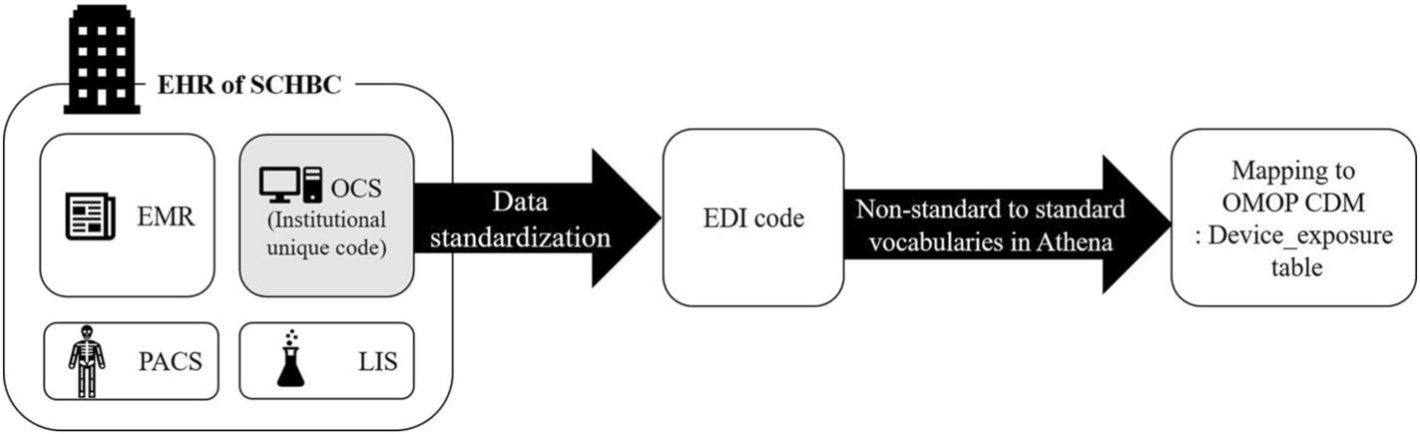
Current CDM mapping process of medical device data in Korea and scopes of this study. Among medical device data recorded in each medical institution's EHR, the institutional unique code recorded in the OCS is converted into a national insurance electronic data interchange (EDI) code. EDI codes are mapped using an OMOP CDM standard vocabulary. The standard vocabulary for medical device data is SNOMED. *EHR* electronic health records, *CDM* common data model, *SCHBC* Soonchunhyang University Bucheon Hospital, *OCS* online charting system, *EDI* electronic data interchange, *EMR* electronic medical record, *PACS* picture archiving and communication system, *LIS* laboratory information system, *MDV* medical device vigilance.

Soonchunhyang University Bucheon Hospital (SCHBC) in Bucheon, Gyeonggi Province, Republic of Korea is covered by Federated E-health Big Data for Evidence Renovation Network (FEEDER-NET)^13,14^. According to the user manual provided by FEEDER-NET^15^, institutional unique prescription codes were used as medical device data sources and converted into electronic data interchange (EDI) codes used by national insurance claims in Korea. The EDI code is registered in Athena. It can be entered into the CDM as it is. However, since it is a non-standard term, it is converted to the standard term, Systemized Nomenclature of Medicines—Clinical Terms (SNOMED-CT), and entered into the CDM if possible (Fig. 1). Global Medical Device Nomenclature (GMDN) terms are the basis of SNOMED CT medical device content^16^.

### Analysis of medical device data recorded in EHR

**Figure 2:**
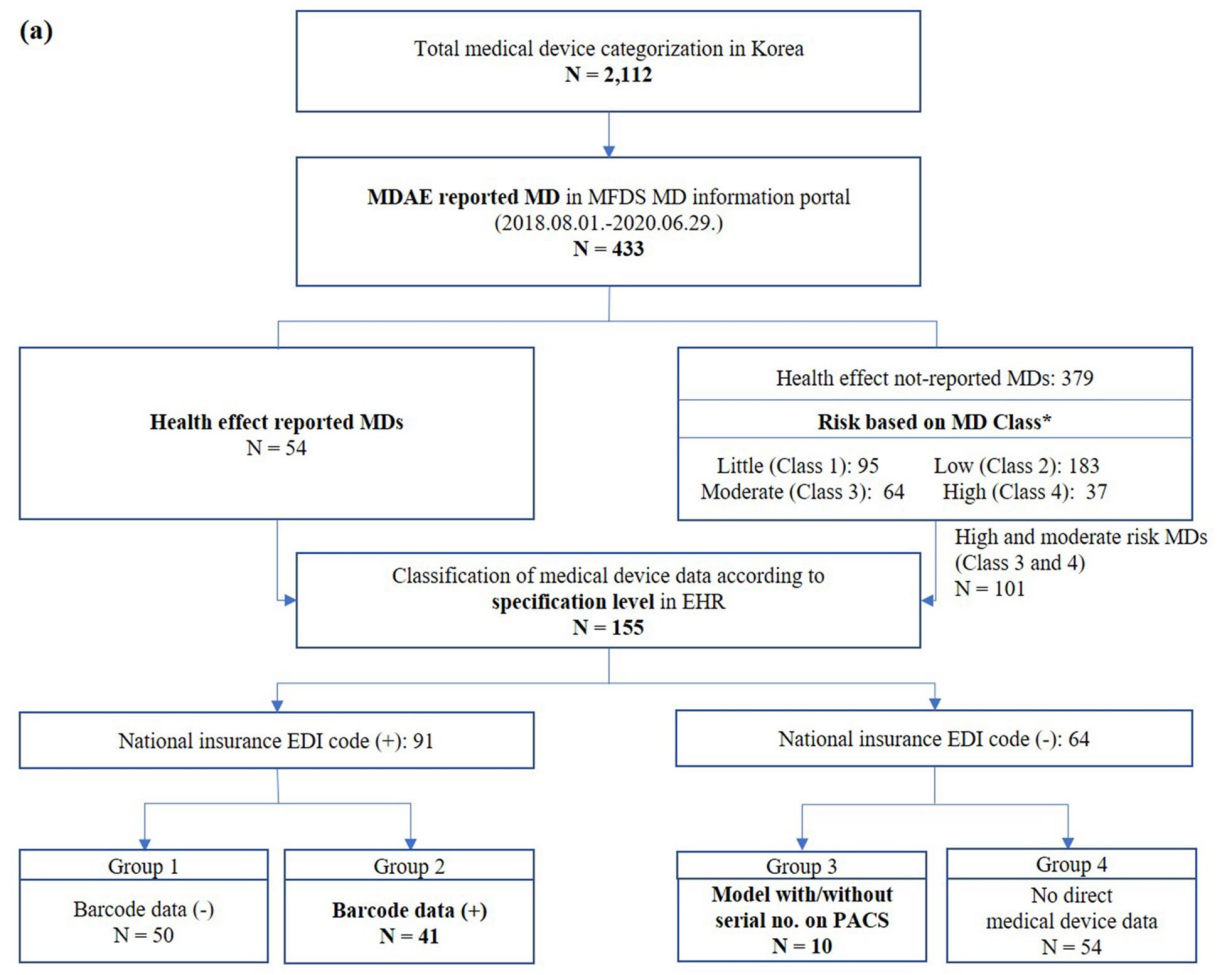

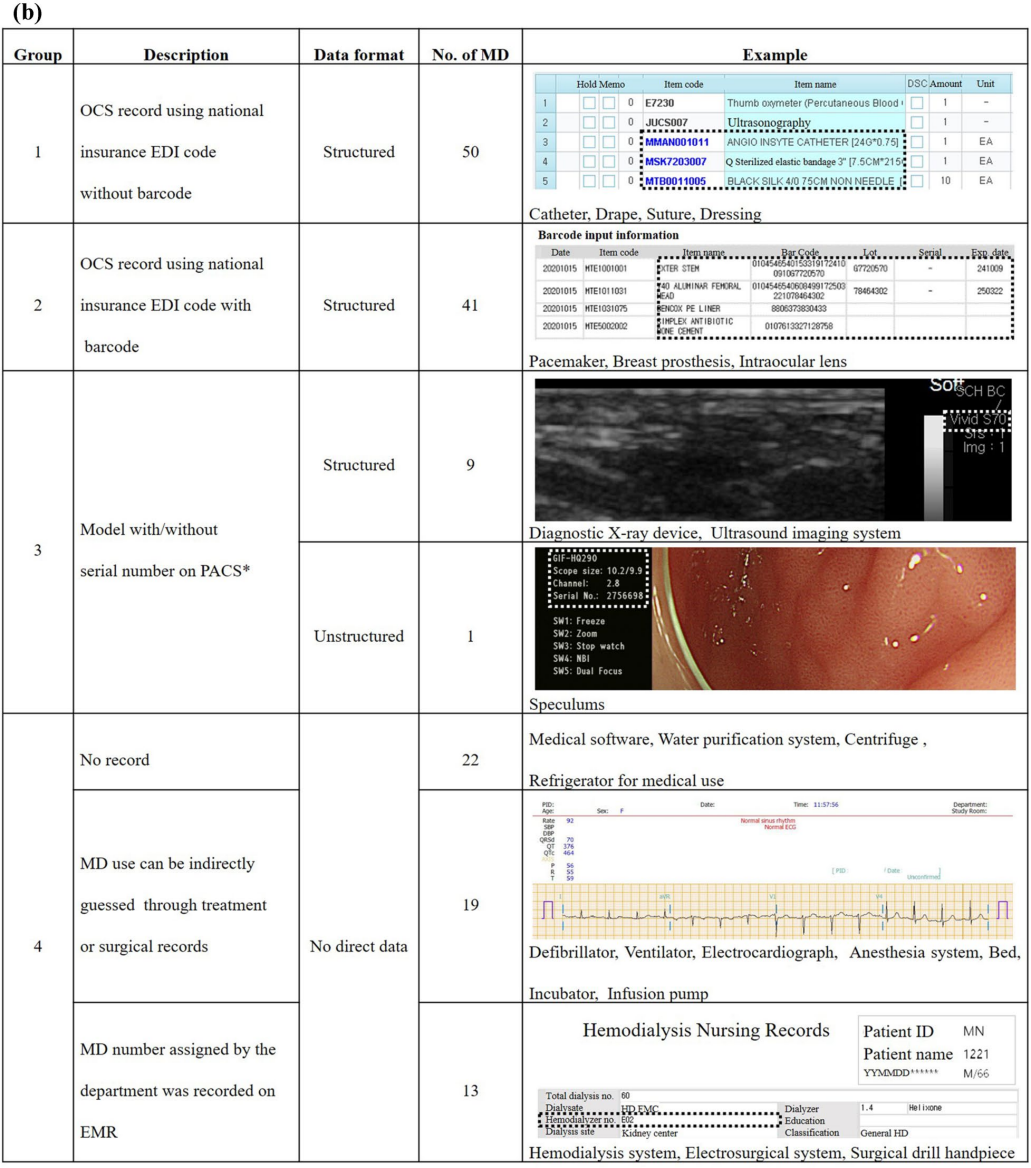
A total of 155 medical devices were finally enrolled for the investigation of medical device data on EHR (**a**). Medical device information EHR was classified into four groups (**b**). *MDAE* medical device adverse event, *MD* medical device, *MFDS* Ministry of Food and Drug Safety, *EHR* electronic health records, *EDI* electronic data interchange, *PACS* picture archiving and communication system, *EMR* electronic medical record.

Among the 2112 medical devices posted by the Ministry of Food and Drug Safety (MFDS), 433 posts had MDAE occurrence. Fifty-five medical devices were reported to have health effects on patients. Of the remaining 379 medical devices whose health effects were not reported, 101 had moderate or high potential risks. A total of 155 medical devices were finally enrolled to investigate medical device data in EHR (Fig. 2a). Medical device information from SCHBC EHR was classified into four groups (Fig. 2b). Ninety-one medical devices (91/155, 58.7%) were prescribed by code (EDI code linked) on online charting system (OCS) when used in patients. Fifty devices without other information recorded other than OCS prescriptions were classified into group 1. Forty-one medical devices (41/155, 26.5%) recorded as barcode data in the surgical nursing record on EMR were classified into group 2. Ten medical devices (10/155, 6.5%) recorded model name on picture archiving and communication system (PACS) were classified into group 3. Nine of them had model names displayed separately from the image without serial number or lot number. The other one, a gastroscope, had model name and serial number of the device displayed as part of the image. Fifty-four medical devices without direct device data were classified into group 4. Twenty-two of them did not have any records. Nineteen of them did not have a direct record. They were assumed to be used medical devices needed for treatment or surgeries. Thirteen had a number assigned by the user in the electronic medical record (EMR). However, they were considered as group 4 because such user given number had been assigned to a new device without any comments when replacing the device.

**Figure 3:**
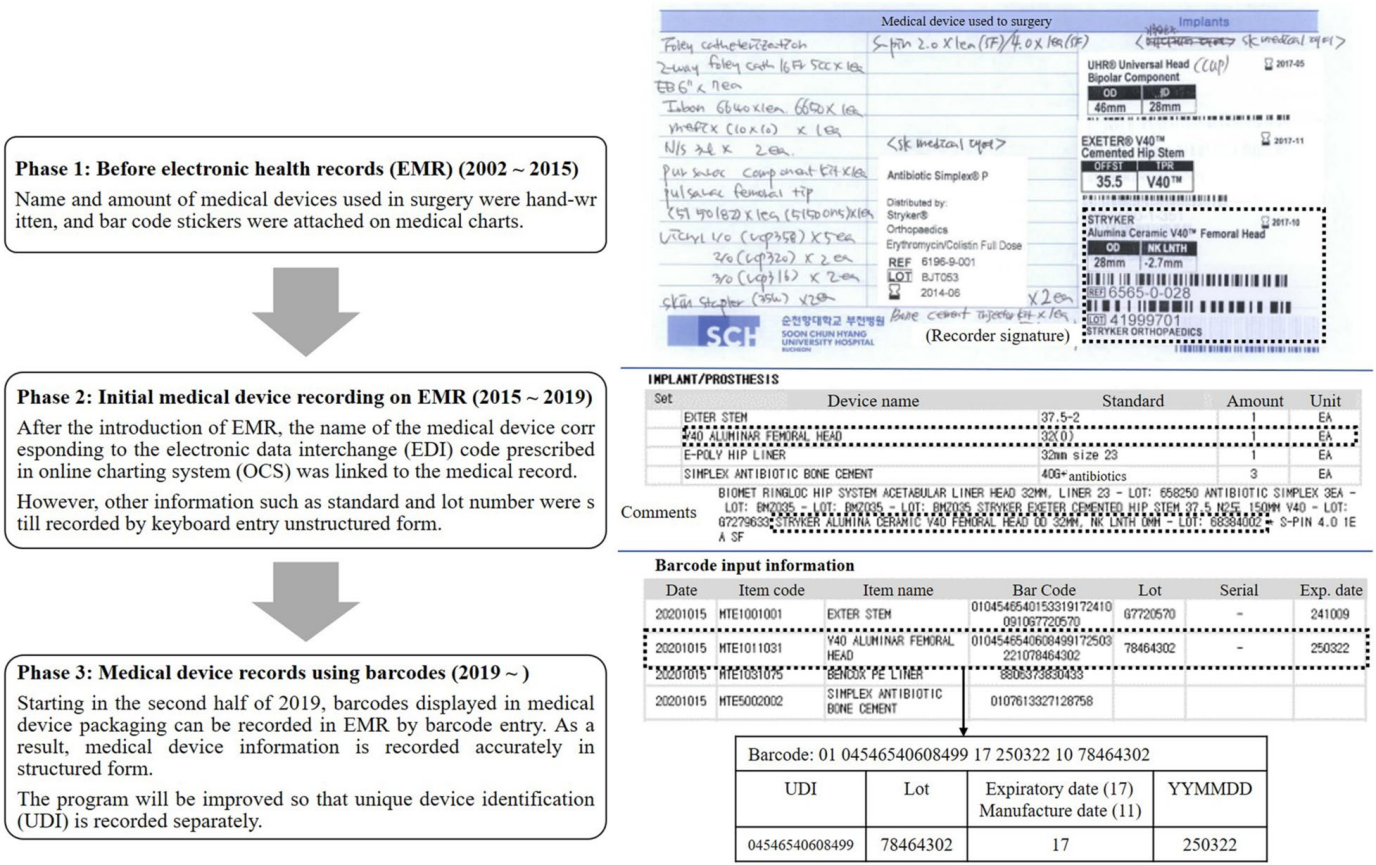
Changes of implanted medical device records in our institution.

Figure 3 shows changes of implanted medical device records in our institution. Prior to the introduction of EMR, OCS prescriptions using the National Insurance EDI code and scan images of barcode stickers for devices were recorded. Initially, the device name was interlinked with the OCS prescription and the barcode data was replaced by a keyboard entry lot number instead of a scan image. In 2019, barcode readers were introduced to improve barcode data recording. When the barcode is read, the entire barcode data and some distinguished data (lot or serial number and expiration date) are entered at a specified location.

### Focus analysis of medical device data

**Figure 4:**
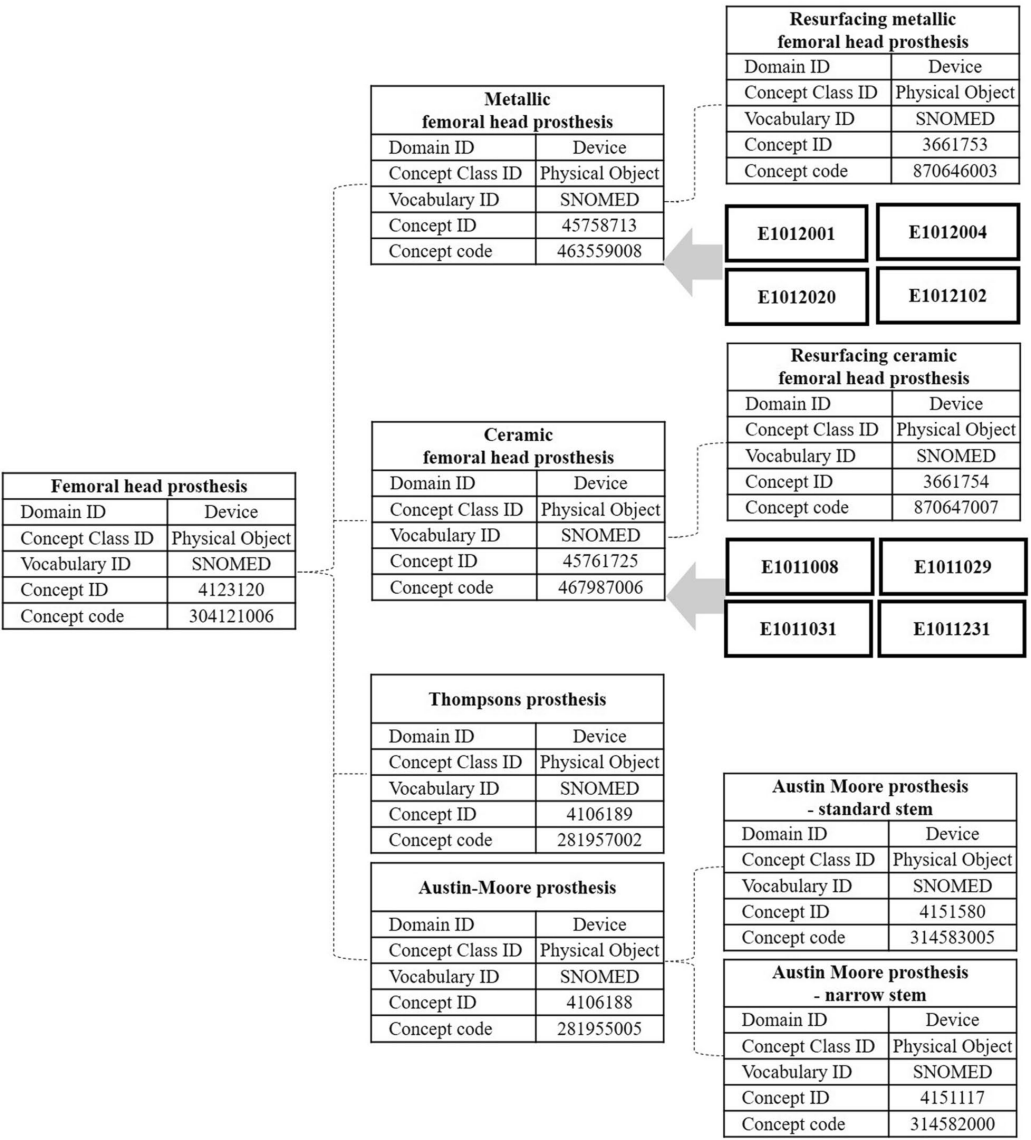
Femoral head hierarchy of SNOMED-CT. Only three of 11 EDI codes registered on the SCHBC CDM are mapped to the concept ID 45761725, while the remaining eight are mapped to concept ID 4558713 or 45761725 depending on the material. *CDM* common data model, *SCHBC* Soonchunhyang University Bucheon Hospital, *EDI* electronic data interchange, *SNOMED-CT* systematized nomenclature of medicine clinical terms.

National insurance has 31 and 28 codes for metal and ceramic femoral heads, respectively. There were a total of 11 codes registered in our institute's OCS (Table 1). On the CDM of SCHBC, three of those 11 femoral head codes were mapped to the standard concept of SNOMED-CT (code mapping was provided as Supplementary Table S1), while the other eight EDI terms were mapped to the non-standard concept of SNOMED-CT as shown in Fig. 4.

**Table 1:** List of 11 national insurance EDI codes registered in our institute and their concept ID used to map to the CDM.

National insurance EDI code		SCHBC CDM			
Code	Code name	Concept_ID	Name	Vocabulary	Record count
E1012001	Osteonics femoral head (Howmedica Osteonics Corp, Mahwah, NJ, US)	42097997	Osteonics femoral head	EDI	0
E1012004	VerSys femoral head (Zimmer, Inc, Warsaw, IN)	42103020	VerSys femoral head	EDI	6
E1012020	ic-head (Implantcast GmbH, Buxtehude, Germany)	42094220	ic-head	EDI	10
**E1012102**	**Exeter femoral head (Howmedica Osteonics Corp, Mahwah, NJ, US)**	**42092029**	**Exeter head**	**EDI**	**177**
E1011008	Ceramic head (DePuy Synthes Companies, Marwah, Indiana, US)	42089833	Ceramic head	EDI	84
E1011029	Ceramic ball head (Plus Orthopedics AG, Rotkreuz, Switzerland)	42089827	Ceramic ball head	EDI	38
**E1011031**	**V40 Alumina femoral head (Howmedica Osteonics Corp, Mahwah, NJ, US)**	**42102799**	**V40 Alumina femoral head**	**EDI**	**334**
E1011231	Biolox Delta Ceramic V40 femoral head (Howmedica Osteonics Corp, Mahwah, NJ, US)	42088730	Biolox Delta ceramic V40 femoral head	EDI	0
E1011023	Femoral head, ceramic (Lima Corporate Spa, Udine, Italy)	45761725	Ceramic femoral prosthesis	SNOMED-CT	35
E1011002	Ceramic head (Howmedica Osteonics Corp, Mahwah, NJ, US)				
E1011204	Ceramic femoral head (CeramTec AG, Plochingen, Germany)				

*CDM* common data model, *SCHBC* Soonchunhyang University Bucheon Hospital, *EDI* electronic data interchange, *SNOMED-CT* systematized nomenclature of medicine clinical terms.

Among metal and ceramic heads, EDI codes E1012102 and E1011031 represent the largest record count (bold).

Both metal and ceramic femoral heads were used most frequently by Howmedica Osteonics Corp (Mahwah, NJ, USA). Thus, they were selected as focus groups: E1012001, E1012102, E1011031, and E1011231. E1011002 was not included as a focus group because it had a prescription record currently excluded from national insurance.

**Figure 5:**
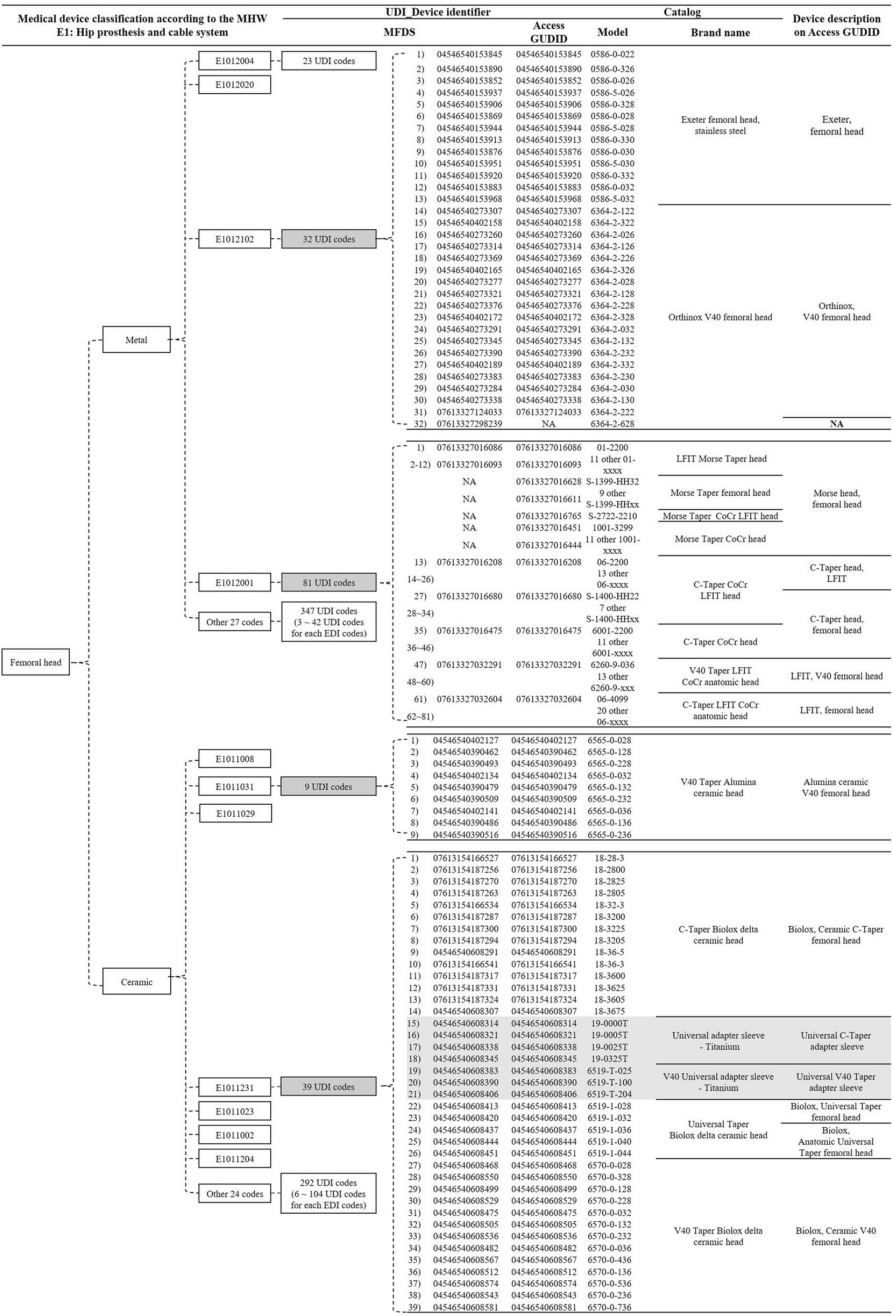
Analysis of various medical device data for the chemical head selected as a focus group. *MHW* Ministry of Health and Welfare, *UDI* unique device identifier, *MFDS* Ministry of Food and Drug Safety, *GUDID* global unique device identification database.

**Figure 6:**
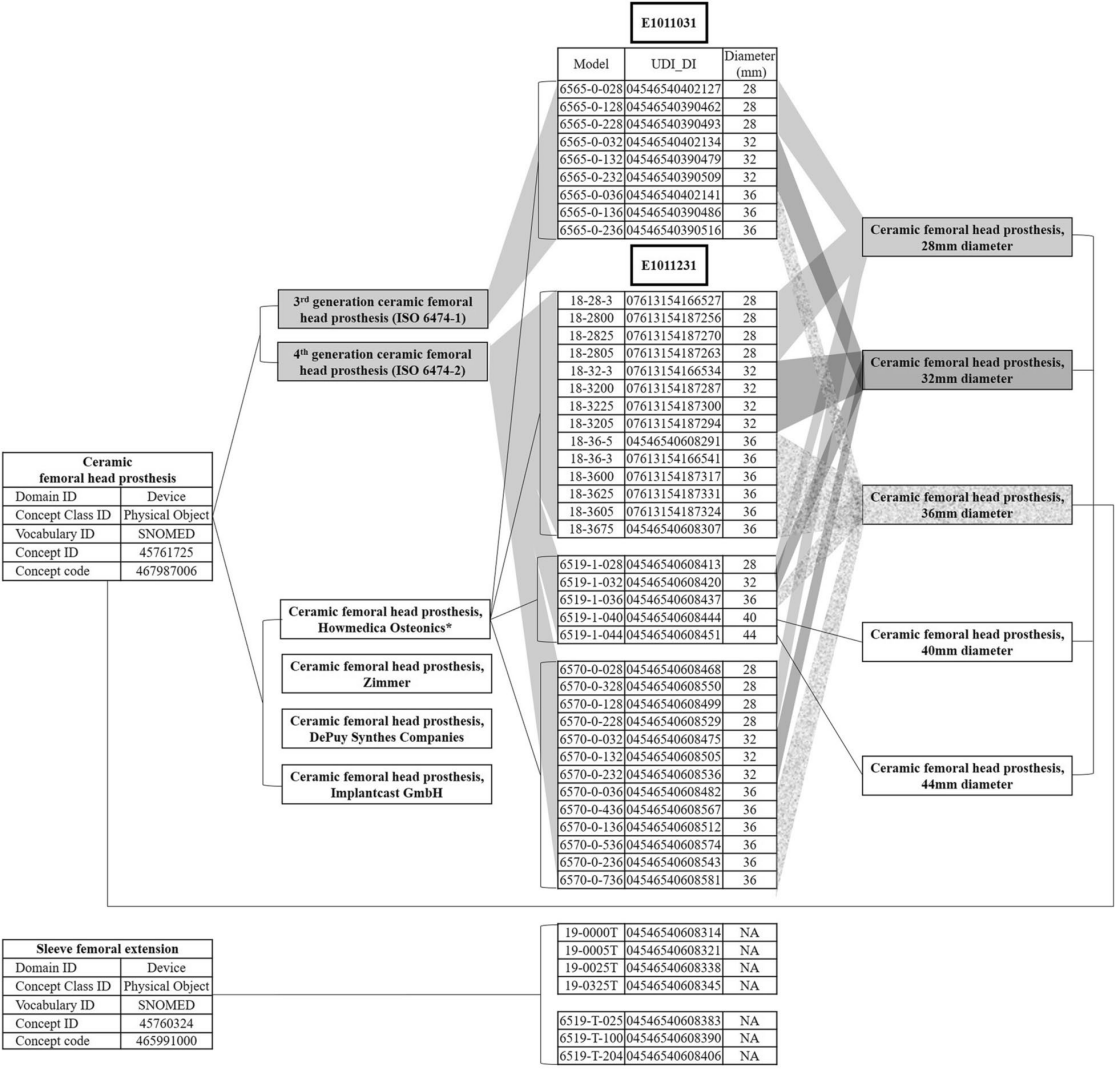
Ceramic femoral head hierrarchy was applied to Stryker's products registered with national insurance EDI codes E1011031 and E1011231. *EDI* electronic data interchange, *SNOMED* systematized nomenclature of medicine clinical terms, *UDI* unique device identifier, *DI* device identifier.

Various medical device data sources used in this study provided different medical device classifications and data (Table 2). The national insurance EDI code was commonly used by MOHW and MFDS data sources. Because EDI codes were applied without distinction to medical device specifications, the four EDI codes selected as focus groups were reclassified using the MFDS's medical device classification criteria. Models were available from the MFDS, the manufacturer’s catalog, and Access Global Unique Device Identification Database (GUDID). EDI data were found from the MFDS and Access GUDID. There were 31 EDI codes registered as metal femoral head in national insurance (Fig. 5). Each of 32 models registered under the E1012102 code was gaved a unique device identifier (UDI). The manufacturer's catalog categorized 32 models as Exter femoral head stainless steel and Orthinox V40 chemical head. The 32nd model (6364-2-628) of the E1012102 was registered with the MFDS as 07613327298239, but was not registered with the Access GUDID. The 81 models registered under the E1012001 code were categorized under five brand names depending on the type of taper (Morse, C and V40) and Low Friction Ion Treatment (LFIT) application. The first model to the 12th model were listed in the catalog as LFIT Morse taper head and Access GUDID as Morse head femoral head. By searching for Morse head femoral head in Access GUDID, 22 additional UDIs were identified that were not in the MFDS. They were classified under three brand names by the manufacturer. The 27th to 34th models were classified as C-Taper CoCr LFIT heads in the catalog, but were classified as C-Taper heads in Access GUDID. There were 30 EDI codes registered as ceramic femoral head in national insurance. The nine models registered with E1011031 code had a single brand name. Thirty-nine models were registered in E1011231, with six models (from 15th to 21st) being adapter sleeves, not femoral heads. The remaining 33 models were classified as three in the catalog and four brand names in the Access GUDID. Figure 6 presents ceramic femoral head hierarchies according to manufacturer, ceramic type, and head diameter. The 48 models registered in codes E1011031 and E1011231 were reclassified for the hierrarchies presented.

**Table 2:** Various medical device data acquired from multiple sources.

	Ministry of Health and Welfare	Ministry of Food and Drug Safety	Manufacturer’s global catalog	Access GUDID
Target device	National insured devices	Devices licensed for sale in Korea	Stryker’s devices on the market	Devices licensed for sale in USA
Category	O (own taxonomy)	O (own taxonomy)	NA	GMDN
Class (1–4)	X	O	NA	NA
License number	X	O	NA	NA
Bander/manufacturer	O	O	O	O
Brand name	O	O or X	O	O
EDI	O	O*	NA	NA
Model	X	O	O	O
UDI	X	O	X	O
Lot number	X	X	X	X

*GUDID* global unique device identification database, *GMDN* global medical device nomenclature, *NA* not available, *EDI* electronic data interchange, *UDI* unique device identifier.

*In case of insurance coverage.

## Discussion

MDV is currently performed through a variety of voluntary and mandatory reporting mechanisms to report events, not rates^17^. The calculation of the latter requires a denominator, that is, the total number of the used devices. For drugs, the situation is different. Sentinel Initiative monitors the safety and comparative effectiveness of drugs by leveraging national drug codes recorded at the point of care or the point of sale and transmitted to payers via insurance claims^18^. In Korea, the MFDS is required to record and preserve usage of some medical devices such as implanted medical devices for more than a year in order to track patients. However, medical institutions tend to write this as a dedicated document for medical management rather than recording it in EMR (personal communication). Mapping these data to an OMOP CDM is hard to expect. Therefore, national insurance EDI code has been used as a medical device data source in OMOP CDM (Fig. 1), similar to the Sentinel Initiative. However, there has been no approach or feasibility assessment on whether CDM established by Korean institutions is suitable for MDV. Based on EHR record and OMOP CDM conversion status of medical device data, this study performed preliminary feasibility assessment of CDM-based MDV.

- Adequacy of medical device EDI code for MDV purpose

As of 2016, 97.1% of Korea's population are covered by national insurance. Health Insurance Review and Assessment Service (HIRA) has developed the EDI code system to classify and identify drugs, medical services, and devices. It also maintains the EDI code system. HIRA mandates the use of EDI vocabulary in the claim system. For this reason, every Korean EHR system uses the EDI vocabulary for most drugs, medical procedures, and devices. It has advantages as a data source in that it is structured and domestic standardized. However, EDI term has not been acknowledged as a standard vocabulary in the way that the Current Procedural Terminology, fourth edition has in the United States because its quality has never been audited^19^. Instead, there were several attempts to map the EDI term to SNOMED-CT^20–22^. Nevertheless, the EDI code as a medical device data source showed several limitations in the present study. First, only a few codes were mapped to standard terms (Table 1). Second, it excluded non-claimed medical devices (groups 3 and 4 in Fig. 2a,b). Third, medical devices might be unintentionally intermixed in a single EDI code even if all EDI codes are mapped to standard terms (Fig. 5).

- Candidates for medical device data source other than EDI code

EHR data capture is critical to device-tracking efforts^23^. For this purpose, the structure of the data and the presence of records on EHR are both important. EHRs are recorded as either unstructured keyboard entry narrative data or structured coded data^24^. Structured data enable the reuse of data collected in the course of clinical care. However, structured data systems are typically difficult to use, time consuming, and often inflexible for expressing complex clinical concepts. Previously reports have indicated that 80% of medical data are unstructured data while only 20% of medical data are structured data used mostly for disease diagnosis^25^. As shown in Fig. 2b, structured medical device data include prescription code modified national insurance EDI code (50 devices), barcode entry (41 devices) including UDI, and model (9 devices) recorded in PACS. Other medical devices without charging patients have been recorded in an unstructured form for management or not recorded. These unstructured data cannot be processed by algorithm directly. Additional data mining, natural language processing, and text analytics would be needed. In the Veteran Administration’s Cardiovascular Assessment, Reporting, and Tracking (CART) initiative using free text and limited structured data entry, a dataset of questionable validity for surveillance efforts has been observed^26^. Tradeoffs between structured and unstructured data recording observed in Fig. 3 complicate the reuse of data. Which medical device data should be captured was discussed considering the existence of EHR records, the structure of the data, and the potential for standardization.

The first option was brand name. It has advantages of easy verbal communication. However, it is not recorded in EHR. Considering the discrepancy in brand name recorded in the manufacturer’s catalog and Access GUDID with only a few brand names registered in the MFDS (Table 2), it is considered difficult to standardize brand names and record them in EHR. It was predicted that CDM conversion of brand name level might increase the loss of data integrity. In pharmacovigilance, brand name level CDM conversion showed 6 to 7% information loss to drug exposure^8,9^. The second option was model (catalog number). The model is familiar to users as it is recorded in the catalog as well as in the MFDS. It gives specifications, enabling detailed classification of medical devices. The International Consortium of Orthopaedic Registries (ICOR)^27^ is using models of devices for their identification. However, there has been no worldwide consensus on the encoding of part numbers^28^. Different prostheses have been identified with the same model and different models have been used for the same prosthesis. In addition, variations in models within and between different companies’ products are possible. For example, in Fig. 5, the LFIT Morse taper head is named 01-xxx. However, Morse taper femoral head is named S-1399-HHxx, although the two have the same manufacturer. Additional EHR records would be required to use the model because only 10 medical devices’ model were recorded in our EHR (Fig. 2a, group 3). The last option was UDI. UDI includes device identifier (UDI-DI) and production identifier (UDI-PI). The UDI-DI should be globally unique^29^. It should identify the specific version or model of a device and its manufacturer. The UDI-PI identifies one or more of the following when present on the label of the device: the lot or batch within which a device was manufactured, the serial number, the expiration date, and the date on which a specific device was manufactured^30^. Manufacturers must label medical devices with UDI code in a machine-readable format, such as a barcode, radiofrequency identifier (RFID), and human-readable text. The United States Food and Drug Administration (FDA) released a proposed rule in July 2012 that medical devices distributed in the US should be fitted with UDI^31^. Manufacturers must submit key product to GUDID that can be accessed by both patients and providers^32^. The European Union (EU) medical device regulations also require fitting of UDI to all medical devices sold in the EU. This requirement came into effect from 2015 to 2019^33^. European Database on Medical Devices (EUDAMED) is expected to have UDI coding^34^. In Korea, mandatory UDI attachment was implemented by the MFDS in July 2019. All medical devices should have UDI attachment by 2022. To efficiently manage them, an integrated information system for medical device is also in operation^35^. Although it is introduced after 2019, UDI is recorded in the EHR as a barcode entry in our institute (Fig. 3). It can be easily structured as it is generated in accordance with regulations. Therefore, the authors determine that UDI is the most appropriate medical device data source to be mapped to CDM.

Health systems may benefit from UDI adoption for several reasons other than postmarket surveillance. The UDI establishes a standard identifier to track the device that can be captured by barcode scanning already widely used for many purposes^36^. Scanning this single UDI barcode is easier than individually capturing several fields, such as manufacturer, product type, and serial number. It can improve inventory management systems to monitor inventory, enable automatic reordering, and ensure that devices are used before their shelf life expires^37^. Moreover, knowledge of a patient's implanted devices or those used previously can improve clinical care. If issues are identified or the device is recalled, they could be notified quickly^38^. As with barcode e-MAR (electronic drug administration records), which significantly reduces medication errors, barcode scanning in medical devices can improve patient safety^39^. For UDI adaption, hospitals need to upgrade their EHR and supply-chain management programs to capture UDIs and train staff. Although some large health care systems have successfully implemented UDI for implantable devices^26^, UDI has had limited effect because it is available in neither EHR nor claims^38^. The Office of the National Coordinator for Health Information Technology has ruled that EHRs must have the capability to record UDIs to receive certification. However, this capability is not mandatory^18^. Requiring the UDI in claims is expected to prompt EHR integration^32^. It has the potential to greatly enhance postmarket surveillance and provide essential data for performing research using real-world evidence. CMS has previously expressed concern about such a requirement because the cost and complexity of changing operating and technical systems to include the UDI in claims. The current proposal shortens claims requirement to include only UDI-DI, meaning that specific models could be identified if UDI-PI is not be available. A partial implementation that uses only UDI-DI could decrease the utility of the UDI because important information about a faulty device's production may be missing^38^. Additionally, even if all providers modify their EHR related to claims and if payers modify their claims-processing systems to accept UDIs, MDV system will not automatically emerge. In the MDV system, some unique characteristics of medical devices should be considered^40^. Medical devices consist of multiple components with complex interactions among devices. The effect of device-specific learning^41^ and differences in experience between providers can influence clinical outcomes^42^. Mechanical failure, even software error, could be a potential cause of failure. None of these issues is usually a problem with drugs^7,43,44^. A functioning medical device evaluation system would be needed.

Although UDI can make unique identification of medical device possible^45^, it is only useful for identifying an issue with a specific device from one manufacturer. Generic device grouping of UDI would enable systematic problem to be identified early. Global Medical Device Nomenclature (GMDN) code fulfils requirements of a generic device group^46^. The GMDN is a list of generic. It was specified as the naming convention for the device portion of the UDI by FDA. The Preferred Term in GMDN is the valid description of a group of devices. It does not differentiate between device models or those from different manufacturers with the same intended use and technology. Therefore, GMDN system enables early identification of a systematic problem not limited to one manufacturer, but shared by other products that use the same technology or materials. In SNOMED-CT system, the only medical device standard vocabulary considered in OMOP CDM, the concept of device is mapped to a subset of GMDN codes. However, various analyses were considered difficult with stratification of SNOMED-CT. Work to expand the GMDN content in SNOMED-CT is required to satisfy wider purposes of SNOMED-CT linked to the use of medical devices. To support its further development, developing medical device descriptions in clinical terminology, linking to additional clinical data, and adding more layers of more specific medical device terms would be needed^16^. For example, the authors identified two EDI codes from ceramic femoral heads as examples of UDI for medical devices and sub-grouped them into materials, manufacturers, and specifications based on opinions of orthopedic experts (Fig. 6).

This study has several limitations. First, since a single institution status was investigated, the situation might be different for other countries or institutions. Second, only representative devices in each medical device category were analyzed. Therefore, the EHR recording status for devices not directly identified might differ from this study, even if they are included in the target medical device category.

OMOP CDM, which is built in our institution, uses EDI code as a medical device data source. Despite its advantages of structural and standardization, EDI is matched in one-to-many with UDI, including devices classified as different GMDNs. Therefore, it is difficult to ensure the accuracy of surveillance results if all medical devices corresponding to a single EDI code are considered to have a single characteristic. To communicate safety information of medical devices, it is recommended to use internationally accepted UDI rather than EDI code as a data source. To introduce UDI, each institution needs to have a barcode reader, develop EHR, and train users. To compensate for the vulnerability of UDI to systematic problem identification, analysis of generic device groups using GMDN is necessary. In this respect, SNOMED-CT that reflects GMDN is appropriate for CDM-based MDVs. However, since analysis according to various device characteristics is difficult with current classification, adding more specific layers is essential. Finally, even if UDI is recorded in EHR and converted to CDM, additional MDV systems are needed to analyze it.

## Methods

We conducted a cross-sectional study at Soonchunhyang University Bucheon Hospital (SCHBC) in Bucheon, Gyeonggi Province, Republic of Korea. This study was approved by the Institutional Review Board of SCHBC. Informed consent was waived (SCHBC 2020-08-028-001).

### Analysis of medical device data recorded in EHR

To evaluate the adequacy of EDI for MDV purposes and to determine the existence of medical device data other than EDI, the target medical device was selected and medical device records of EHR were grouped.

Selection of target medical devices: According to the Ministry of Food and Drug Safety (MFDS) notice in May 2020, medical devices in Republic of Korea are classified into 2112 categories. The following three criteria were applied to select medical devices to evaluate the frequency of MDAE and their risk to patients:

1. Medical devices with MDAEs posted on the MFDS medical device information portal. MDAEs reported in the Republic of Korea have been posted on the MFDS medical device information portal since October 2016 (https://udiportal.mfds.go.kr/). Since posts before August 2018 did not include details of MDAE, this study was conducted using information posted after 2018.
2. Medical devices with patients’ health effect caused by MDAE posted on the portal.
3. Although MDAEs posted on the portal did not show their effects on patients’ health, medical devices were classified as moderate (class 3) or high (class 4) potential risk according to the classification of medical devices in the Republic of Korea.

According to data format, data were classified as unstructured narrative text and structured coded data^47^. The number and percentage of medical devices corresponding to each information level and data formet were obtained. Microsoft Excel 2019 (Microsoft, Redmond, WA, USA) was used for statistical processing.

### Focus analysis of medical device data

To provide a detailed example of medical device data, focus group analysis was conducted using single item: femoral head. Medical device data related to the focus group were retrieved and collected from the MOHW's medical device price list notice^48^, the MFDS's Information Portal^49^, and the U.S. national library of medicine's Access GUDID^50^.

To analyze SNOMED-CT conversion status of medical device data, Observational Health Data Sciences and Informatics (OHDSI) open-source software and OMOP CDM version 5.3 database were used. EHR of SCHBC was converted to source name of cdmpv531_0920_bucheon. Central vocabulary service Athena (http://athena.ohdsi.org) was used to assess the hierarchy of SNOMED-CT.

### Ethics approval

This study was approved by the Institutional Review Board of Soonchunhyang University Bucheon Hospital (SCHBC) (approval number: SCHBC 2020-08-028-001).

### Consent to participate

Informed consent was waived by the IRB.

## Supplementary Information

Supplementary Information.

## Data Availability

Data are available from the corresponding author upon reasonable request.
