# Supplementary material for: Preliminary feasibility assessment of CDM-based active surveillance using current status of medical device data in medical records and OMOP-CDM
Source: Sci Rep. 2021 Dec 15;11:24070. doi: 10.1038/s41598-021-03332-6 (PMC8674329; doi:10.1038/s41598-021-03332-6)
Supplement: Supplementary file 1 — Supplementary Information. [file 41598_2021_3332_MOESM1_ESM.pdf]

Supplementary Table S1. Code mapping table of Femoral heads

Source_ domain_id	Local_cd	Local_cd_name	Target_ concept_id	Target_ domain_id	Original concept_code	Concept_name	Vocabulary_id
Device	E1011002	CERAMIC HEAD 전규격(ZIRCONIA ALUMINA) [HOWMEDICA OSTEONICS]	45761725	Device	467987006	Ceramic femoral head prosthesis	SNOMED
Device	E1011023	FEMORAL HEAD	45761725	Device	467987006	Ceramic femoral head prosthesis	SNOMED
Device	E1011204	CERAMIC FEMORAL HEAD 전규격 (ALUMINUM OXIDE(AL_2O_3)) [CERAMTEC AG]	45761725	Device	467987006	Ceramic femoral head prosthesis	SNOMED
Device	E1011008	CERAMIC HEAD	42089833	Device	E1011008	CERAMIC HEAD	EDI
Device	E1011029	CERAMIC BALL HEAD	42089827	Device	E1011029	CERAMIC BALL HEAD	EDI
Device	E1011031	V40 ALUMINAR FEMORAL HEAD	42102799	Device	E1011031	V40 ALUMINAR FEMORAL HEAD	EDI
Device	E1011231	BIOLOX DELTA CERAMIC FEMORAL HEAD	42088730	Device	E1011231	BIOLOX DELTA CERAMIC FEMORAL HEAD	EDI
Device	E1012001	OSTEONICS FEMORAL HEAD	42097997	Device	E1012001	OSTEONICS FEMORAL HEAD	EDI
Device	E1012004	VERSYS FEMORAL HEAD	42103020	Device	E1012004	VERSYS FEMORAL HEAD	EDI
Device	E1012020	IC-HEAD COCRMO	42094220	Device	E1012020	IC-HEAD COCRMO	EDI
Device	E1012102	PRECISION HEAD	42098841	Device	E1012102	PRECISION HEAD	EDI
